# Underlying and contributing causes of mortality from CDC WONDER—Insights for researchers

**DOI:** 10.1016/j.ahjo.2025.100499

**Published:** 2025-01-10

**Authors:** Abdul Mannan Khan Minhas, Laurence S. Sperling, Sadeer Al-Kindi, Dmitry Abramov

**Affiliations:** a Department of Medicine, Baylor College of Medicine, Houston, TX, United States of America; b Section of Cardiovascular Research, Baylor College of Medicine, Houston, TX, United States of America; c Division of Cardiovascular Medicine, Emory University School of Medicine, Atlanta, GA, United States of America; d Division of Cardiology, Houston Methodist, Houston, TX, United States of America; e Division of Cardiology, Loma Linda University Medical Center, Loma Linda, CA, United States of America

**Keywords:** CDC WONDER, Mortality, Contributing, Underlying

## Abstract

**Background:**

The multiple cause of death files available through the Center for Disease Control and Prevention Wide-ranging Online Data for Epidemiologic Research (CDC WONDER) present underlying and contributing causes of mortality. We sought to evaluate potential differences in mortality reporting that may occur based on utilization of only underlying versus utilization of both underlying and contributing cause of mortality.

**Methods:**

All-cause and top 5 underlying causes of deaths in individuals ≥25 years of age from 2011 to 2019 occurring within United States were extracted from CDC WONDER. Deaths for the top 5 underlying causes of death as underlying and as underlying or contributing causes were extracted. For each cause, we calculated the percentage of the deaths that would be reported if only the underlying versus the underlying or contributing mortality was reported.

**Results:**

Between 2011 and 2019, the top 5 underlying causes of mortality were heart disease, malignant neoplasm, chronic lower respiratory disease, cerebrovascular disease, and accidents. For these causes, the percentages of deaths presented by reporting (underlying)/(underlying or contributing) causes were 53 %, 91 %, 50 %, 59 %, and 79 % respectively.

**Discussion/conclusion:**

Data within the commonly utilized multiple cause of death files from CDC WONDER demonstrate that reliance solely on the underlying cause of mortality may underestimate important contributions of common contributing causes. These findings could aid in the understanding of published research and may shape the framework for future studies utilizing multiple cause of death data through CDC WONDER.

## 1 Introduction

Disease-specific causes or contributors to mortality are important endpoints for epidemiological studies that assess population-level morbidity and mortality. In the United States (US), mortality data from the multiple cause of death files from National Center of Health Statistics as evaluated through the Center for Disease Control and Prevention Wide-ranging Online Data for Epidemiologic Research (CDC WONDER) are often used to evaluate causes of mortality. CDC WONDER provides information regarding underlying or contributing causes of mortality based on International Classification of Disease codes, with heart disease, malignant neoplasms, chronic lower respiratory disease, cerebrovascular disease, and accidents comprising the leading underlying causes of mortality. Data expressed within multiple cause of death files available at CDC WONDER are extrapolated from death certificates which denote single underlying cause of death and up to twenty additional (contributing) causes of deaths [1]. Although the trends and demographics associated with individual mortality causes have been well described, there are limited data on the relationship between underlying and contributing causes of mortality using CDC WONDER. Understanding the relationship between underlying and contributing causes of mortality may help identify the implications of various approaches to classifying mortality, especially given variation in research approaches where only the underlying or both the underlying and contributing causes are used to report the burden and trends of key causes of mortality [2,3]. Therefore, we sought to evaluate the relationship between underlying and total (underlying or contributing) causes of mortality for each of the top 5 mortality causes from multiple cause of death files using CDC WONDER.

## 2 Methods

The top 5 underlying causes of deaths in individuals ≥25 years of age from 2011 to 2019 occurring within the US were extracted from CDC WONDER [4]. The Multiple Cause-of-Death Public Use record death certificates were used to analyze these deaths both as “underlying” or “contributing or underlying” cause of death on nationwide death certificates. The top 5 causes of deaths were: Diseases of heart (I00-I09, I11, I13, and I20-I51), Malignant Neoplasms (C00-C97), Chronic Lower Respiratory diseases (J40-J47), Cerebrovascular diseases (I60-I69). Accidents (Unintentional Injuries) (V01-X59,Y85,Y86). Crude and age-adjusted mortality rates (AAMR) per 100,000 population were determined. Crude mortality rates were determined by dividing the number of deaths by the corresponding U.S. population of that year. AAMRs were determined by standardizing the deaths to the year 2000 U.S. population [5]. We calculated percentages of top 5 underlying cause of deaths over same causes of deaths as underlying or contributing (e.g. 100× [number of heart disease deaths as underlying/number of heart disease deaths as underlying or contributing]). Additionally, we evaluated proportion of underlying cause of cardiovascular disease deaths (I00-I99) over underlying or contributing causes of cardiovascular disease deaths from among cohorts based on age, sex, race and ethnicity, metropolitan-nonmetropolitan areas, state, region, place of death and year to determine potential differences in death reporting. To further explore underlying vs contributing designation via a specific example of two commonly studied conditions, we extracted the data on diabetes mellitus (a chronic condition) and acute myocardial infarction (an acute condition). This was done to determine how frequently each was reported as the underlying cause when both were listed on the death certificate. Institutional Review Board approval was not sought because CDC WONDER contains publicly available, anonymized data.

## 3 Results

Between 2011 and 2019, there were a total of 23,684,167 deaths reported in individuals ≥25 years of age. The number of deaths for the top 5 underlying causes, including the number and AAMR for each cause as the underlying and the underlying or contributing cause, is noted in Table 1. For the top 5 underlying causes of death: heart disease, malignant neoplasm, chronic lower respiratory disease, cerebrovascular disease, and accidents, the percentages of deaths from (underlying)/(underlying or contributing) causes were 53 %, 91 %, 50 %, 59 %, and 79 % respectively. Stated another way, these percentages demonstrate the epidemiologic burden of that cause of mortality that would be reported if only the underlying cause of mortality is evaluated versus the burden that would be reported if both the underlying or contributing cause were evaluated. For example, reporting only the underlying cause of heart disease would identify only 53 % of deaths where heart disease was listed on the death certificate as playing a role in that patient's mortality. To further provide an example of underlying vs contributing designation for common conditions, we extracted the data on diabetes mellitus (chronic disease) and acute myocardial infarction (acute disease). In total, 233,940 death certificates had both diabetes mellitus and acute myocardial infarction listed. Out of these, 125,367 (54 %) had acute myocardial infarction designated as underlying cause of death and 81,609 (35 %) had diabetes mellitus listed as underlying cause of death.

**Table 1:** Total, crude, and age adjusted mortality rates for top 5 underlying causes of mortality as underlying and contributing causes.

	Deaths	Population	Crude rate	Age adjusted rate (95 % confidence interval)	Percent of (underlying)/(underlying+contributing)
All cause AAMR	23,684,167	1,945,248,489	1217.5	1094.7 (1094.2–1095.1)	
Diseases of heart (underlying) AAMR	5,638,267	1,945,248,489	289.8	257.5 (257.3–257.8)	
Diseases of heart (contributing/underlying) AAMR	10,724,337	1,945,248,489	551.3	491.8 (491.5–492.1)	53 %
Malignant neoplasms (underlying) AAMR	5,303,141	1,945,248,489	272.6	242.3 (242.0–242.5)	
Malignant neoplasms (contributing/underlying) AAMR	5,828,310	1,945,248,489	299.6	266.3 (266.1–266.6)	91 %
Chronic lower respiratory diseases (underlying) AAMR	1,365,527	1,945,248,489	70.2	62.8 (62.7–62.9)	
Chronic lower respiratory diseases (contributing/underlying) AAMR	2,705,677	1,945,248,489	139.1	124.2 (124.0–124.3)	50 %
Cerebrovascular diseases (underlying) AAMR	1,242,542	1,945,248,489	63.9	57.2 (57.1–57.3)	
Cerebrovascular diseases (contributing/underlying) AAMR	2,096,922	1,945,248,489	107.8	96.5 (96.4–96.6)	59 %
Accidents (unintentional injuries) (underlying) AAMR	1,191,142	1,945,248,489	61.2	59.8 (59.7–59.9)	
Accidents (unintentional injuries) (contributing/underlying) AAMR	1,514,309	1,945,248,489	77.8	74.7 (74.6–74.9)	79 %

Percentage of deaths with cardiovascular disease classified as (underlying)/(underlying or contributing) causes among cohorts based on age, sex, race and ethnicity, and location of death are shown in Supplementary Tables 1 and 2. There were percentage differences among different cohorts in cardiovascular deaths that were classified as (underlying)/(underlying or contributing). More numerically prominent differences were noted based on location of death, with inpatient medical facilities demonstrating that 51 % of cardiovascular disease were classified as (underlying)/(underlying or contributing) while this value was 70 % for deaths in the medical facilities-outpatient setting or the emergency room and 72 % for those who presented as dead on arrival in medical facilities. There were also numeric differences based on US State, with the percentage of deaths with cardiovascular disease listed as the (underlying)/(underlying or contributing) ranging from 49 % in Minnesota to 64 % in the District of Columbia.

## 4 Discussion

This analysis on the relationship between underlying and contributing causes from multiple cause of death files through CDC WONDER demonstrates several important findings. A reliance solely on the underlying cause of mortality may lead to under-appreciation of the burden of a particular disease as a contributor to mortality. There were differences among key cohorts in the percentage of deaths from cardiovascular mortality that were classified as (underlying)/(underlying or contributing). These results may guide interpretation of published studies, facilitate design of future studies of mortality data from CDC WONDER, and may help aid future efforts to optimize mortality data collection in the US.

In the US, cause of death information is primarily provided by physicians or medical examiners/coroners based on circumstances [6]. Immediate cause of death, underlying cause of death, and contributing causes are recorded [7,8]. Based on the information provided in part I of the death certificate, underlying cause of death is determined by automated software or expert coders [9,10]. Compiled state data are eventually transferred to National Center for Health Statistics and can be accessed through CDC WONDER. There are limited prior data on the association between contributing and underlying causes of mortality in the US. Prior US National Center for Health Statistics data from the early 2000s demonstrated variability between disease states regarding classification as underlying or contributing causes of mortality, and noted that reliance solely on underlying cause of mortality may underestimate the mortality burden for several diseases [11]. We expand on these findings with data from the current era and for the top causes of mortality, and also demonstrate potential variability in reporting underlying versus underlying or contributing causes of death among key cohorts including US States. Multiple causes of death captured modest additional burden for some diseases like malignant neoplasms (9 %) and accidents (21 %), but substantially greater burden for other diseases including heart disease (47 %), cerebrovascular diseases (41 %) and chronic lower respiratory diseases (50 %). Therefore, utilizing both the contributing and underlying cause results in a more comprehensive assessment of a particular cause of mortality. For example, we demonstrated that when diabetes mellitus and acute myocardial infarction are both co-listed, either of them could be potentially attributed as the underlying cause, which points towards heterogeneity in practice of attributing the cause of death as underlying and may reduce the presumed accuracy of limiting analysis only to an underlying cause. Our results highlight that classification of mortality solely with an underlying cause may lead to under-recognition of contribution to mortality by several conditions, limiting public health efforts to appreciate and subsequently target the burden of these key causal contributors to mortality.

Further heterogeneity is noted when evaluating underlying versus underlying or contributing causes based on subgroups, including variability in classification based on place of death and US State. As mortality data are an integral part of public health efforts to track the impact of key lethal diseases, additional efforts to standardize the mortality report processes are important to ensure the accuracy of the data used for public health decision making. Moreover, comparing the number of deaths from CDC WONDER, either with underlying or underlying/contributing causes, with epidemiological estimates obtained from other sources or datasets may also provide further insights into the accuracy of the various approaches.

Consequently there is increasing recognition of the importance of evaluating multiple causes of mortality [[12], [13], [14]] as well as various statistical approaches to better assess multiple causes of mortality [15]. Current data suggest that there is an average of 3 causes of mortality listed per death certificate, with approximately 20 % listing only one cause and 45 % listing 3 or more causes [16]. Hence, it is likely that many of the contributing causes listed would have important contribution to mortality. Furthermore, around a third of death certificates in one US State were noted to have only one cause of mortality listed [17], therefore potentially missing important contributing causes. Currently, the CDC WONDER allows one to query maximum of two contributing disease and one underlying disease at one time. Expanding the capabilities of the online system to incorporate more diseases along with improvement of data collection may provide researchers with more opportunities to effectively analyze the epidemiological trends.

Multiple cause of death files accessed through CDC WONDER have important limitations which are worth noting. The underlying and contributing causes in this dataset rely on accurate reporting by the physician at the time of completing the death certificate. There may be intrinsic limitations to death certificate data [18]. Causes of mortality may be difficult to determine and it may be plausible that certain kind of conditions, particularly chronic conditions, may be under-reported on death certificates.

Additionally, the severity of conditions listed on the death certificate for underlying or contributing causes is not available. Variables about health status, treatment, associated health conditions, and socioeconomic factors are not available. The mortality data used in this analysis was obtained from CDC WONDER, although additional microdata are available through the National Center for Health Statistics [19]. Nevertheless, the CDC WONDER is commonly used in research studies despite known limitations and our analysis may provide important insight for researchers working with these data.

In conclusion, our analysis of the cause of death files using CDC WONDER demonstrates the relationship between underlying and total (underlying or contributing) causes of mortality for the top 5 causes of mortality in the US. Researchers should keep in mind that utilizing only underlying compared to the contributing or underlying cause may lead to under appreciation of contribution to mortality for the diseases being studied. Additional efforts appear warranted to standardize death reporting the US. These results can provide insight to researchers working with CDC WONDER data and potentially guide future efforts to optimize mortality data collection in the US.

## CRediT authorship contribution statement

**Abdul Mannan Khan Minhas:** Writing – review & editing, Writing – original draft, Formal analysis, Conceptualization. **Laurence S. Sperling:** Writing – review & editing. **Sadeer Al-Kindi:** Writing – review & editing. **Dmitry Abramov:** Writing – review & editing, Writing – original draft, Supervision.

## Ethics statement

We submit that:

• the work described has not been published previously except in the form of a preprint, an abstract, a published lecture, academic thesis or registered report.
• the article is not under consideration for publication elsewhere.
• the article's publication is approved by all authors and tacitly or explicitly by the responsible authorities where the work was carried out.
• if accepted, the article will not be published elsewhere in the same form, in English or in any other language, including electronically without the written consent of the copyright-holder.

## Declaration of competing interest

The authors declare that they have no known competing financial interests or personal relationships that could have appeared to influence the work reported in this paper.

## Appendix A Supplementary data

Supplementary tables Image 1
