# Supplementary material for: Underlying and contributing causes of mortality from CDC WONDER—Insights for researchers
Source: Am Heart J Plus. 2025 Jan 10;50:100499. doi: 10.1016/j.ahjo.2025.100499 (PMC11782113; doi:10.1016/j.ahjo.2025.100499)
Supplement: Supplementary file 1 — Supplementary tables [file mmc1.docx]

Supplementary Table 1: Percentage of Underlying over Underlying or Contributing Causes for Cardiovascular Disease among key cohorts

|  |  | Underlying | Underlying or contributing |  |
| --- | --- | --- | --- | --- |
|  |  | Deaths | Deaths | Percentage |
| Overall |  | 7439451 | 13085048 | 56.85 |
| 25-44 years |  | 161224 | 313717 | 51.39 |
| 45-64 years |  | 1240501 | 2254735 | 55.02 |
| 65-79 years |  | 2037292 | 3834056 | 53.14 |
| 80+ years |  | 4000434 | 6682540 | 59.86 |
| Male |  | 3771772 | 6598051 | 57.16 |
| Female |  | 3667679 | 6486997 | 56.54 |
| NH-White |  | 5839472 | 10219205 | 57.14 |
| NH Black or African American | | 909442 | 1577368 | 57.66 |
| NH Asian or Pacific Islander | | 185334 | 331090 | 55.98 |
| NH American Indian or Alaska Native | | 37417 | 76110 | 49.16 |
| Hispanic or Latino | | 443930 | 841802 | 52.74 |
| Nonmetropolitan | | 1425677 | 2515970 | 56.67 |
| Metropolitan | | 6013774 | 10569078 | 56.90 |
| Northeast |  | 1398167 | 2466519 | 56.69 |
| Midwest |  | 1710211 | 2917581 | 58.62 |
| South |  | 2871028 | 4973085 | 57.73 |
| West |  | 1460045 | 2727863 | 53.52 |
| Medical Facility - Inpatient | | 2090004 | 4090867 | 51.09 |
| Medical Facility - Outpatient or ER | | 779587 | 1113992 | 69.98 |
| Medical Facility - Dead on Arrival | | 47568 | 66165 | 71.89 |
| Decedent's home | | 2198147 | 3709740 | 59.25 |
| Hospice facility | | 396238 | 707660 | 55.99 |
| Nursing home/long term care | | 1566721 | 2792479 | 56.11 |
| 2011 |  | 781117 | 1351755 | 57.79 |
| 2012 |  | 785154 | 1361812 | 57.66 |
| 2013 |  | 798743 | 1395118 | 57.25 |
| 2014 |  | 805554 | 1406790 | 57.26 |
| 2015 |  | 834308 | 1457516 | 57.24 |
| 2016 |  | 838482 | 1473345 | 56.91 |
| 2017 |  | 856964 | 1522191 | 56.30 |
| 2018 |  | 866584 | 1548931 | 55.95 |
| 2019 |  | 872545 | 1567590 | 55.66 |

Supplementary Table 2: Percentage of Underlying over Underlying or Contributing Causes for Cardiovascular disease among States

|  | Underlying | Underlying or contributing |  |
| --- | --- | --- | --- |
| State | Deaths | Deaths | Percentage |
| Minnesota | 99756 | 203692 | 48.97 |
| Nebraska | 41851 | 84143 | 49.74 |
| Rhode Island | 26589 | 51727 | 51.40 |
| California | 751853 | 1457351 | 51.59 |
| Connecticut | 82494 | 157620 | 52.34 |
| Colorado | 85342 | 162844 | 52.41 |
| Vermont | 15198 | 28880 | 52.62 |
| Oregon | 85806 | 162631 | 52.76 |
| North Dakota | 16655 | 31140 | 53.48 |
| West Virginia | 57993 | 107492 | 53.95 |
| Washington | 135568 | 251049 | 54.00 |
| South Carolina | 121288 | 221699 | 54.71 |
| Oklahoma | 114620 | 209503 | 54.71 |
| Alaska | 9442 | 17213 | 54.85 |
| Massachusetts | 139917 | 254513 | 54.97 |
| Texas | 507185 | 922569 | 54.98 |
| Kentucky | 118325 | 214786 | 55.09 |
| Mississippi | 90726 | 164040 | 55.31 |
| South Dakota | 20767 | 37474 | 55.42 |
| New Hampshire | 29325 | 52909 | 55.43 |
| Indiana | 166015 | 298986 | 55.53 |
| Georgia | 211200 | 378461 | 55.80 |
| New York | 488565 | 875456 | 55.81 |
| New Mexico | 43040 | 76651 | 56.15 |
| North Carolina | 223246 | 393519 | 56.73 |
| Idaho | 33428 | 58732 | 56.92 |
| Ohio | 326197 | 572489 | 56.98 |
| Arizona | 139055 | 243990 | 56.99 |
| Maine | 33637 | 58594 | 57.41 |
| Wyoming | 11748 | 20433 | 57.50 |
| New Jersey | 212430 | 368362 | 57.67 |
| Hawaii | 30848 | 53378 | 57.79 |
| Arkansas | 90535 | 155461 | 58.24 |
| Iowa | 82220 | 140519 | 58.51 |
| Montana | 24603 | 42018 | 58.55 |
| Wisconsin | 137082 | 233586 | 58.69 |
| Tennessee | 181069 | 308519 | 58.69 |
| Utah | 41909 | 71298 | 58.78 |
| Kansas | 69846 | 116943 | 59.73 |
| Pennsylvania | 370012 | 618458 | 59.83 |
| Florida | 539631 | 900347 | 59.94 |
| Maryland | 134629 | 223486 | 60.24 |
| Delaware | 23079 | 38134 | 60.52 |
| Virginia | 169998 | 280546 | 60.60 |
| Nevada | 67403 | 110275 | 61.12 |
| Michigan | 283658 | 458273 | 61.90 |
| Illinois | 298635 | 475390 | 62.82 |
| Alabama | 149626 | 236930 | 63.15 |
| Missouri | 167529 | 264946 | 63.23 |
| Louisiana | 123155 | 194738 | 63.24 |
| District of Columbia | 14723 | 22855 | 64.42 |
